# Sleep parameters improvement in PTSD soldiers after symptoms remission

**DOI:** 10.1038/s41598-021-88337-x

**Published:** 2021-04-23

**Authors:** P. F. Rousseau, R. Vallat, O. Coste, H. Cadis, F. Nicolas, M. Trousselard, P. Ruby, S. Khalfa

**Affiliations:** 1 Laboratoire de Neurosciences Cognitives UMR 7291, Aix Marseille Université CNRS, Marseille, France; 2 Lyon Neuroscience Research Center, Brain Dynamics and Cognition Team, INSERM UMR 1028, CNRS UMR 5292, Université Claude Bernard Lyon 1, Lyon, France; 3 Center for Human Sleep Science, Department of Psychology, University of California, Berkeley, CA 94720-1650 USA; 4 Hôpital d’Instruction des Armées Desgenettes, Unité de Pathologie du Sommeil, Lyon, France; 5 Service de Psychiatrie, Hôpital d’Instruction des Armées Sainte-Anne, Toulon, France; 6 Unité Neurophysiologie du Stress, Département des Neurosciences et des Contraintes Opérationnelles, Institut de Recherche Biomédicale des Armées, Brétigny sur Orge, France

**Keywords:** Psychiatric disorders, Stress and resilience, Non-REM sleep, REM sleep, Sleep

## Abstract

Eye movement desensitization and reprocessing (EMDR) is a psychotherapy for the treatment of posttraumatic stress disorder (PTSD). It is still unclear whether symptoms remission through EMDR therapy is associated with a beneficial effect on one of the PTSD symptoms, sleep disturbance. Our objective was therefore to study sleep parameters before and after symptom remission in soldiers with PTSD. The control group consisted of 20 healthy active duty military men who slept in a sleep lab with standard polysomnography (PSG) on two sessions separated by one month. The patient group consisted of 17 active duty military with PTSD who underwent EMDR therapy. PSG-recorded sleep was assessed 1 week before the EMDR therapy began and 1 week after PTSD remission. We found that the increased REMs density after remission was positively correlated with a greater decrease of symptoms. Also, the number of EMDR sessions required to reach remission was correlated with intra-sleep awakenings before treatment. These results confirm the improvement of some sleep parameters in PTSD after symptoms remission in a soldier's population and provide a possible predictor of treatment success. Further experiments will be required to establish whether this effect is specific to the EMDR therapy.

## Introduction

Sleep disturbances represent a key factor in the genesis and maintenance of psychiatric disorders^1^. An estimated 90% of people with posttraumatic stress disorder (PTSD) report difficulty initiating and maintaining sleep^2,3^. Even in the absence of nightmares, sleep problems remain the most frequently reported complaint in PTSD^4^. Self-reported poor sleep quality is only minimally explained by psychiatric comorbidity, age, and sex in these patients, which suggests an important role of the syndrome in the sleep impairment^5^.

The severity of PTSD symptoms are positively correlated with sleep disturbances^6^. Sleep problems may in turn worsen daytime PTSD symptoms^7^, and contribute to comorbid psychiatric disorders such as major depression^8–11^, suicidal behaviour^12^, and substance abuse^13^. Sleep disturbances in PTSD are also correlated with a decreased ability to carry out daily activities^14–16^, again fitting with problems related to insufficient sleep in the general population, including functional impairment, reduced quality of life^17–22^, and increased risk of accidents^23^. Of note, sleep problems do not always improve after otherwise successful treatment for PTSD^4,24–26^.

Polysomnography (PSG) studies have yielded inconsistent findings regarding the presence and nature of sleep disturbance in individuals with PTSD^27^, with some studies reporting reduction and/or disruption of rapid eye movement (REM) sleep^28–30^, whereas others have described a higher percentage of REM sleep^31–34^ in individuals with PTSD compared with other clinical groups or good sleepers without PTSD. A recent study that used at-home sleep recordings in 71 control subjects and 60 military-related PTSD subjects found a significantly reduced percentage of REM sleep in the latter group^35^. Inconsistent results have been found for non-REM (NREM) sleep, with some studies reporting PTSD-associated NREM disturbances^35–38^, and several others reporting no effects^31,39,40^. Based on these findings, meta-analyses identified a core pattern of sleep disruption in PTSD^1,41^, consisting of longer N1 sleep, shorter N2 and N3 sleep, as well as greater rapid eye movements (REMs) density in REM sleep compared to individuals without PTSD. Nevertheless, more studies are needed to better characterize the core sleep disturbances in PTSD patients.

The mechanisms and neurophysiological correlates of the attenuation or resolution of such disorders by pharmacological^42^ or psychotherapeutic^4,43^ treatments are still poorly understood. Increased central nervous system adrenergic activity might contribute to the pathophysiology of PTSD^44,45^ by interfering with normal REM sleep^46^, so studies have investigated the use of effective pharmacological treatments^42^, including prazosin, a generic alpha-1 adrenergic antagonist, in military veterans with PTSD. Objective sleep assessment studies involving prazosin and a placebo have shown significantly increased sleep time and REM sleep duration, reduced REM sleep latency^47^ and no changes in NREM sleep or sleep onset latency. Cognitive behavioral therapy for insomnia also increased total sleep time in PTSD individuals compared to a wait-list group^4^, and both prazosin and imagery rehearsal therapy improved sleep quality and decreased nightmare frequency^48^.

Eye movement desensitization and reprocessing (EMDR) therapy has been shown to significantly decrease PTSD symptoms and is therefore among the first-line psychological interventions for PTSD^49,50^. Beyond a significant decrease in PTSD symptoms, EMDR has shown its capacity to modify the brain anatomy after remission^51,52^ or the brain’s functional metabolism to the traumatic event script^53,54^. Yet, its precise effect and mechanism remain unclear. To date, only one study has investigated the effect of EMDR therapy on sleep using PSG^43^. They compared mood, anxiety, autonomic activation, as well as subjective and objective sleep parameters before and after EMDR therapy in a group of 13 PTSD patients and 11 healthy controls. Before treatment, PTSD patients had significantly lower subjective sleep quality and an increase PSG-defined wake after sleep onset (WASO) compared to the control group. These parameters were however non-significantly different from the control group after treatment, which suggests that EMDR therapy was associated with sleep improvements in these patients. Even though the authors did not find any significant interaction group × time for the other sleep parameters (most likely because of a low sample size), they did find that the density of REMs during REM sleep was lower after treatment in the PTSD group (Cohen d = 1.37 of within-group comparison for PTSD patients). The latter finding of therapy-induced changes in REMs density is consistent with Stickgold’s theory that the eye movements in EMDR therapy would reproduce the rapid eye movements of REM sleep, and would thus trigger some of the brain mechanisms that are typical of REM sleep^55^, a stage of sleep known for its role in emotional regulation and memory processing. In this theory, the state and eye movements induced by the bilateral alternating stimulation would permit the integration of traumatic memories into associative cortical networks without interference from hippocampally mediated episodic recall. This mechanism would thus alleviate the need for or replace the emotional regulation at play during REM sleep which may induce a diminution in REM sleep REMs density.

Although EMDR therapy can use stimuli other than eye movements (tactile or auditory), recent findings have shown accumulating evidence of a treatment gain of using eye movements as opposed to no eye movements or other forms of bilateral stimulations^56–59^. Accordingly, it is possible that EMDR therapy somehow emulates, and/or compensates for, REM-sleep mechanisms that are supposedly failing in patients with PTSD (as indicated by the high prevalence of nightmares in these patients). Since only few studies so far have objectified the effect of psychotherapies on PTSD patients’ sleep, our study proposes to objectively assess sleep thanks to polysomnography measures before and after PTSD symptoms remission using EMDR therapy. The patient and control groups are from the same population (soldiers) and larger than in previous studies, the patient group is homogenous in origin of trauma (war), sleep has been recorded in an ecological situation (at home), and the sleep parameters have been assessed thanks to an automatic and thus objective method of sleep scoring^60^. Our objectives are to demonstrate and characterize improvement of sleep parameters after PTSD symptoms remission in a soldier's population and to investigate whether some pre-treatment sleep parameters could be predictive of the duration/success of the EMDR therapy used.

Our first goal was therefore to study the effect of PTSD symptoms remission on a wide range of objective sleep parameters in soldiers with PTSD as compared with healthy military controls. Our second objective was to test whether some of these sleep parameters before treatment could predict the efficiency of EMDR therapy in individuals with PTSD, as measured by the number of EMDR sessions needed to achieve remission.

Building on previous findings^4,43,47^, we hypothesized that remission from PTSD would be associated with improvements in objective sleep parameters, and we therefore aimed to provide an exhaustive overview of these sleep parameters, looking not only at sleep macro-structure but also sleep microstructure (e.g. sleep spindles, REMs) as well as spectral power in pre-defined frequency bands.

## Materials and methods

We recruited exclusively male soldiers, divided into two groups: control and EMDR (for participants with PTSD). The justification for including only male soldiers is mainly practical: there are very few female soldiers present in the fighting unit of the French Army, and women in the military are therefore less at risk of being exposed to traumatic experiences.

### Participants

#### Control group

The control group consisted of healthy active duty soldiers recruited by the Institute of Naval Medicine of the Army Health Service in Toulon, France. Individuals selected for the control group had no notable medical history and no current or chronic diseases, no history of depression or alcohol, tobacco or substance abuse or dependence. No participant had any major sleep disorder. Controls were not receiving any medication and had no history of recent shift work or transmeridian travel for at least 2 months before the experiment.

#### EMDR group

Participants were recruited by two psychiatrists in Sainte-Anne military hospital in Toulon, France. The diagnosis of PTSD was established according to the *Diagnostic and Statistical Manual of Mental Disorders*, 4th Edition, Text Revision (DSM-IV TR)^61^. We excluded individuals with present and past neurological or psychiatric conditions, with the exception of anxiety and depressive disorders if their occurrence was related to PTSD. Participants could keep their psychotropic medication only if they took selective serotonin reuptake inhibitors (SSRIs) as long as the therapy did not change during the trial and if the onset of treatment was more than 3 months ago. We also excluded individuals with a history of sleep disorder (assessed objectively or reported by the participant) before the traumatic event. Participants had to have no history of recent shift work or transmeridian travel for at least 2 months before the experiment. Diagnoses were established and clinical interviews were performed by psychiatrists not otherwise engaged in the study. Participants were assessed by two psychiatrists for PTSD and other mental health disorders by using the structured Mini-International Neuropsychiatric Interview^62^ to check for the absence of a psychiatric disorder before the trauma in PTSD and to screen for potential comorbid psychiatric disorders.

All soldiers had PTSD related to war and five took SSRIs. Comorbidities included major depression (9/15), panic disorder (2/15), agoraphobia (12/15) and obsessive–compulsive disorder (1/15); none had an addictive disorder. None had received exposure therapy or cognitive-behavioural therapy before EMDR procedure.

Participants completed the questionnaires listed below, before and after the end of EMDR therapy. PTSD symptoms were evaluated with the Posttraumatic Stress Checklist Scale (PCLS)^63^ and the Clinician-Administered PTSD Scale (CAPS)^64^. Subjective sleep quality was measured using the Pittsburgh Sleep Quality Index (PSQI)^65,66^; and the PSQI-Addendum for PTSD (PSQI-A), which is specific to sleep disorders in PTSD^67,68^. To assess PTSD comorbidity, we used the Beck Depression Inventory^69^. All questionnaires were validated in French.

### EMDR procedure

EMDR therapy involves associations of cognitive, emotional and physical assessments of actual distress to traumatic scenery as well as imaginal exposure while attending to bilateral alternating stimulations^70^. The individual is asked to visualize the most salient aspect of a traumatic memory while the therapist induces bilateral stimulation (by means of ocular, tactile or auditory stimulations). This process results in a change in cognitive processing of memory and cessation of trauma-related distress, such as negative emotion disappearance, while eliminating physical discomfort associated with the initial memory and establishing a positive cognition about the self^70^. The EMDR therapy was performed according to the standard protocol^71^ by one psychiatrist and one psychologist trained and accredited by EMDR Europe. Sessions were planned every 7 to 15 days according to the availability of patients and the therapist.

All traumatic targets related to the traumatic event were treated until reaching a subjective unit of discomfort of zero and completely true positive cognitions without any body discomfort. Hence, at the end of the protocol, participants were symptom-free and no longer had a diagnosis of PTSD after the EMDR therapy, as assessed again by two psychiatrists according to DSM-IV criteria.

### Polysomnography data collection

#### Control group

The sleep data for the control group were collected for a previous published study^72^. In this study, control military participants spent two nights with a delay between two nights of 1 month in the lab to record baseline sleep before performing a simulated flight in a hypobaric chamber. People in the control group were synchronized with diurnal activity (07:00–22:00 h ± 30 min) and nocturnal rest (22:00–07:00 ± 30 min). They slept on a camp bed in total darkness during a fixed time slot i.e. from 22:30 to 06:30. Only data for the nights before the simulated flight were analyzed and compared to the EMDR group. Sleep architecture was assessed from standard polysomnographic recordings including electroencephalography (EEG), electro-oculography (EOG) of each eye and chin electromyography (EMG) by using A10 loggers (EmblaTM, Flaga, Reykjavik, Iceland)^73^. Details of the methodology for this group were previously published^72^.

#### EMDR group

Sleep was recorded before and after treatment at the participant’s home. The first sleep recording took place the week before EMDR began (T0) and the last one the week after therapy ended (T1). EEGs (Cz-A2 and Pz-A2) and EOG of each eye were recorded using a tone Actiwave device (Actiwave, Camntech Ltd.,)^74^ with a 128-Hz sampling frequency and 10-bit resolution.

For recording, electrodes were placed at about 8 pm, then a calibration procedure was launched, and the recorded session was programmed to start at 10 pm. The scalp and skin were prepared with Nuprep abrasive paste. Then the electrodes were fixed with EC2 fixing and conductive paste. In the morning, the participants removed the electrodes and the devices were returned to the experimenters for EEG sleep analysis.

Of importance, participants did not undergo an adaptation night, mostly for issues of feasibility and acceptability to participants. However, sleep recordings were conducted in a natural at-home setting, using a miniature ambulatory system (with only a limited number of channels), thus minimizing any sleep disturbances caused by an unfamiliar environment or a cumbersome recording system.

### Polysomnography data analysis

Sleep PSG recordings of both groups were analyzed using the ASEEGA software^75^. The calculation of sleep parameters was based on an automatic sleep staging algorithm (ASEEGA, v1.3, Physip, France)^60^, which uses a single central EEG channel to identify sleep stages, calculate summary statistics, and perform more advanced analyses such as the calculation of epoch-by-epoch spectral power in determined frequency bands, as well as the detection of several graphoelements of the sleep microstructure (e.g. spindles, alpha bursts in REM sleep). This algorithm has been extensively validated in healthy individuals (accordance rate with manual scorings 82.9%)^60,76^ and patients with sleep or psychiatric disorders^77–80^. The automatic analysis provided by ASEEGA software involves three steps: preprocessing, analysis and classification. This analysis pipeline was developed by Physips, France^60,75^.

Briefly, preprocessing includes downsampling of the raw signal to 100 Hz and automatic artefacts detection^60^. Next, data-driven automated tuning of the frequency bands of interest is performed to take into account the strong inter-individual variability of EEG signals^60^. Finally, the EEG signal is filtered by using a non-uniform filter bank at the previously identified frequency bands. During analysis, the pre-processed signal is analyzed independently within each frequency band of interest. Depending on the type of EEG features to be estimated, autoregressive modelling, Fourier transform, or instantaneous frequency measurement is used to extract spectral and temporal information and detect sleep microstructures (e.g. spindles and alpha bursts). This analysis step also includes rough temporal localization of awakenings and REM episodes. During classification, because of the EEG variability, the use of predefined sleep-stage patterns is ill-suited to automatic sleep scoring. ASEEGA uses an adaptive fuzzy logic iterative system to repeatedly update the sleep stage pattern definitions. The analysis provides values related to the duration of each phase of sleep (N1, N2, N3, REM and Wake) and the latencies of falling asleep and reaching each phase (with sleep onset defined as the first three epochs of N1 sleep or the first epoch of any other sleep stage). It also provides data on sleep microstructures, namely, spindles and alpha burst (average and total duration, average and total power for each).

REMs density in REM sleep was extracted from the two EOG recordings for each individual and at each time by using the automatic method described in (Vallat et al.)^81^ and implemented in the Sleep module of the Visbrain package^82^. The EOG data were missing for two EMDR participants and one control, who were therefore not included in further analyses. For the remaining participants, the following steps were applied. First, the EOG data were visually inspected by an expert scorer (RV) and participants with too many artefacts on both EOGs at one or both times were excluded from further analyses. This step resulted in excluding three patients and three controls. Then, the EOG data were downsampled to 100 Hz and bandpass-filtered between 0.3 and 30 Hz. For each electrode, the signal was smoothed by using a moving average with a window of 200 ms and the detection signal was defined as the absolute of the first derivative (50-ms step) of the smoothed signal. Saccades were detected as events exceeding the mean plus two times the standard deviation of the detection signal, considering only REM sleep epochs. The REMs density was then defined as the total number of saccades (average of the two eyes) divided by the duration of REM sleep in minutes. For four EMDR participants, one of the two EOGs had poor quality data (e.g., the EOG electrode fell off during the night). In those cases, we therefore duplicated the REM density values of the good EOG to the bad EOG. In other words, we assumed that both EOGs had the same REM density values, which were calculated only from the EOG with good data quality. As a final outlier detection step, we computed for each participant the difference in REM density at T1 minus T0. Participants with values above or below 2 SDs on both eyes were excluded. This step resulted in the exclusion of one control participant. In total, our final sample size for REMs analysis was composed of 14 controls and 12 EMDR participants.

We list below all the standard sleep parameters that were included in subsequent analyses^73^. First, standard parameters related to sleep duration and macrostructure such as: the total sleep time (TST), sleep period time (SPT), sleep onset latency (SOL, with sleep onset defined as the first 3 epochs of N1 or the first epoch of any other sleep stage), wake after sleep onset (WASO), sleep efficiency (SE, defined as TST divided by the total recording time), sleep maintenance efficiency (SME, defined as TST divided by SPT), duration and percentage relative to TST of each sleep stage (N1, N2, N3, REM sleep), latency from sleep onset to first epoch of REM sleep, latency from lights out to first epoch of N2 sleep, number of awakenings and density of awakenings per hour of SPT, number of stage shifts and density of stage shifts per hour of SPT, number and duration of REM periods. Second, parameters obtained from spectral analyses, including the relative power in the delta frequency band (0.1–4 Hz) in N3 sleep, N2 sleep and REM sleep, the relative power in the sigma frequency band (12–16 Hz) during N2 sleep, and the relative power in the theta frequency band (4–8 Hz) during REM sleep. Relative powers were calculated by dividing the absolute power in the frequency band of interest by the total power in the 0.1–50 Hz band. Note that we did not include all possible combinations of frequency band and sleep stages (5 frequency bands × 5 sleep stages = 25 parameters), but instead focused on a priori relevant sleep stages and frequency bands, especially in the context of memory consolidation and emotional regulation. Third, parameters measured using automatic detection of the sleep microstructure, such as the number of spindles, their average duration and frequency, and their density per minute of NREM sleep (N2 + N3), the number of alpha bursts during REM sleep and the density of alpha bursts per minute of REM sleep, and similarly, the number and density of REMs during REM sleep.

### Statistical analysis

Patients’ clinical data before and after symptoms remission were compared by two-sided paired *t-*tests and between-group comparisons of clinical and socio-demographic data using two-sided independent *t*-tests. Sleep parameters were analyzed using two-way repeated measures ANOVA with Group (EMDR or Control) as a between factor and Time (T0 or T1) as a within factor. In the event of significant interactions (Group x Time), uncorrected two-sided *t*-tests and/or paired *t*-tests were used for post-hoc comparisons. For the patient’s group, pairwise Pearson correlations were calculated between the sleep parameters and the number of EMDR sessions required to reach remission. All statistical analyses were performed using the Pingouin 0.3.3 package for Python (https://pingouin-stats.org/) created by Vallat, 2018, with the exception of age-adjusted ANCOVA, which were conducted using the JAMOVI software (https://www.jamovi.org/).

### Ethics

The study protocol was in line with the Declaration of Helsinki and was reviewed and approved by the local ethics committee (Comité de Protection des Personnes South Mediterranean 2, registration number 2014-002126-12), and all participants provided written informed consent.

## Results

Our control group consisted of 20 healthy active duty military (mean age 29.4 ± 4.55 years). One of them was not included in subsequent analyses because PSG data was not usable at T1. The EMDR group consisted of 17 active duty military participants with PTSD (mean age 36.8 ± 5.70 years). Two participants—one who did not complete the EMDR therapy, and another whose PSG recording before therapy was not usable—were not included in the analyses. EMDR participants underwent a mean of 4.41 ± 2.09 EMDR sessions (range 2–8) before reaching remission. As shown in Table 1, EMDR therapy significantly reduced symptoms related to PTSD (as measured on the PCLS, CAPS and BDI clinical scales), and significantly improved subjective sleep quality (PSQI and PSQI-A).

**Table 1:** Socio-demographic and clinical characteristics of individuals with post-traumatic stress disorder receiving eye movement desensitization and reprocessing (EMDR) therapy and controls.

	EMDR (n = 17)		Control (n = 20)	*p*-value
**Socio-demographic characteristics**				
Age (years)	36.8 ± 5.70		29.4 ± 4.55	**< 0.001**
Time between T0 and T1 (months)	3.61 ± 1.16		1	**< 0.001**
Duration of illness (years)	6.45 ± 0.92		/	
Duration of therapy (number of sessions)	4.41 ± 2.09		/	
**Use of psychiatric medications**				
Selective serotonin reuptake inhibitors	5/15			
**Psychometric scales**	**T0**	**T1**		
PCLS	63.24 ± 8.68	24.06 ± 7.89		**< 0.001**
CAPS	80.94 ± 14.07	15.25 ± 13.45		**< 0.001**
Beck Depression Inventory	15.65 ± 5.16	3.19 ± 2.74		**< 0.001**
PSQI	12.0 ± 2.76	5.62 ± 3.28		**< 0.001**
PSQI-A	8.82 ± 2.43	2.53 ± 2.20		**< 0.001**
**Comorbidity on the MINI**	**T0**	**T1**		
Major depressive disorder	9/15	0/15		**0.005**
Panic disorder	2/15	0/15		0.16
Agoraphobia	12/15	2/15		**0.005**
Obsessive compulsive disorder	1/15	0/15		0.33

Values are mean ± SD. Significant p-values are in bold.

*PCLS* Posttraumatic Stress Checklist Scale, *CAPS* Clinician-Administered PTSD Scale, *PSQI* Pittsburgh Sleep Quality Index, *PSQI-A* PSQI-Addendum for PTSD, *MINI* Mini-International Neuropsychiatric Interview.

**Figure 1:**
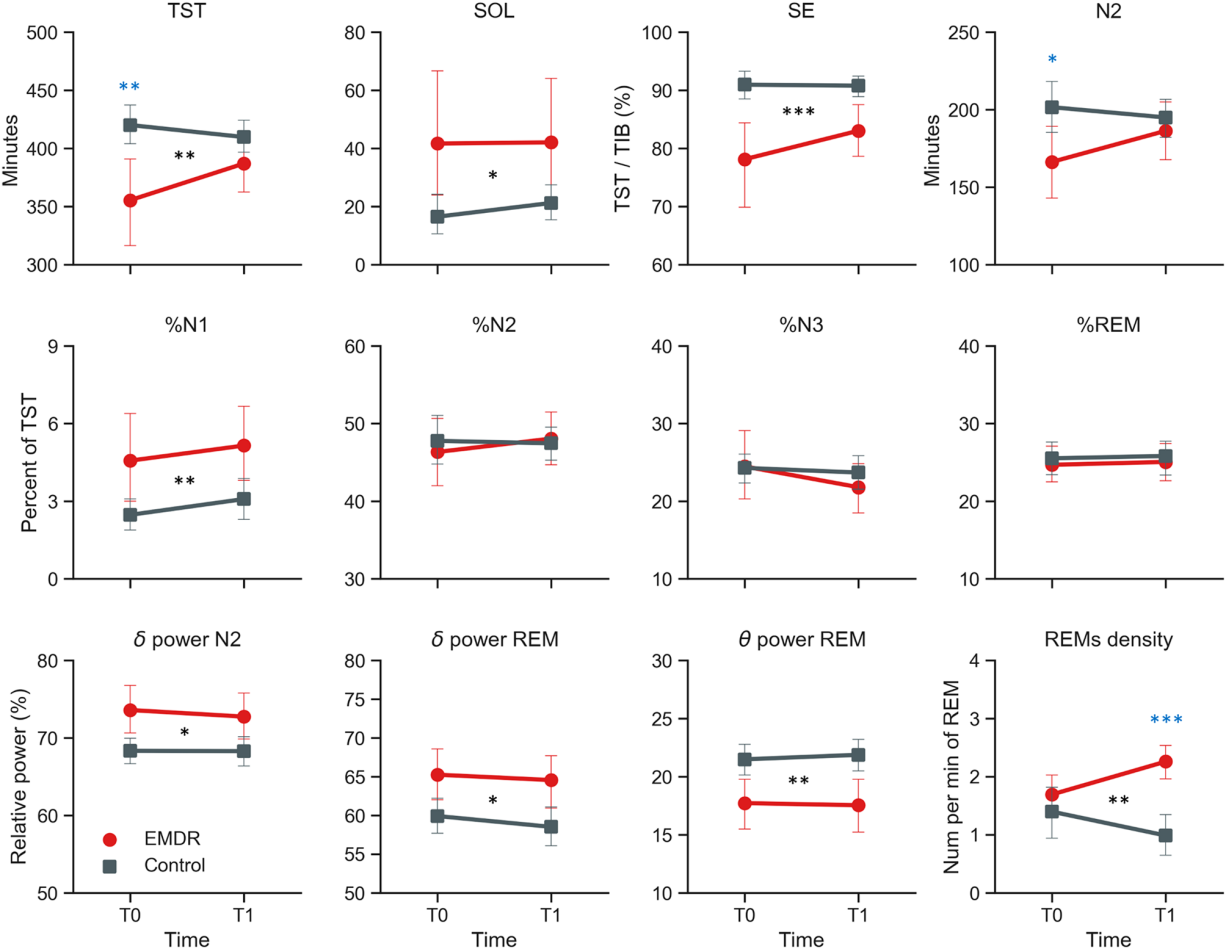
Sleep parameters. Red lines = EMDR group, grey lines = control group. Black stars indicate significant group main effects, while blue stars represent significant post-hoc tests when a significant interaction group × time was present. F-values are reported in Table 2. *p < 0.05, **p < 0.01, ***p < 0.001. *TST* total sleep time, *SOL* sleep onset latency, *SE* sleep efficiency.

The sleep parameters for both groups and both sessions are presented in Fig. 1, Table 2 and S1. We found a significant Group × Time interaction for 4 sleep parameters: total sleep time (TST; F(1, 32) = 5.08, p = 0.031), duration of N2 stage (F(1, 32) = 5.29, p = 0.028), density as well as absolute count of rapid eye movements (REMs) in REM sleep (averaged across both EOG channels; density: F(1, 25) = 8.26, p = 0.008), count: F(1, 25) = 5.23, p = 0.03). Post-hoc tests showed that TST and N2 were significantly higher in the control group than in the EMDR group at baseline (T0, p = 0.009 and p = 0.035 respectively) but not at T1 (both p’s > 0.14). The density and absolute count of REMs were significantly higher at T1 in the EMDR group compared to the control group (p < 0.001 and p = 0.004 respectively), with no such difference at T0 (both p’s > 0.32).

**Table 2:** Sleep parameters.

Group	Control		EMDR		ANOVA (F-values)		
Time	T0	T1	T0	T1	Group	Time	Interaction
TST	419.98 ± 38.0	409.63 ± 30.6	355.31 ± 83.5	386.88 ± 52.7	11.62**	1.19	5.08*
SPT	442.12 ± 35.4	424.63 ± 34.3	387.22 ± 99.3	412.09 ± 47.3	5.3*	0.18	4.11
SOL	16.45 ± 16.4	21.18 ± 13.6	41.62 ± 44.0	42.03 ± 41.5	5.43*	0.79	0.05
WASO	15.60 ± 22.0	10.37 ± 9.2	24.62 ± 21.4	18.47 ± 13.7	3.29	3.02	0.2
SE	90.94 ± 6.0	90.76 ± 4.2	78.11 ± 15.1	83.01 ± 9.3	15.25***	1.22	1.83
SME	95.05 ± 5.2	96.54 ± 2.3	92.54 ± 6.5	93.79 ± 5.1	4.84*	1.72	0.02
N1	10.22 ± 5.5	12.68 ± 7.3	16.47 ± 14.8	19.94 ± 11.9	7.19*	1.63	0.12
N2	201.50 ± 40.0	194.87 ± 30.1	166.19 ± 52.6	186.25 ± 39.8	3.44	1.39	5.29*
N3	101.65 ± 19.4	96.18 ± 17.6	83.50 ± 26.4	83.31 ± 23.6	7.55**	2.72	0.78
REM	106.60 ± 21.3	105.89 ± 22.8	89.16 ± 28.9	97.38 ± 26.4	4.58*	0.86	0.87
%N1	2.47 ± 1.4	3.09 ± 1.8	4.56 ± 3.6	5.14 ± 3.0	9.38**	1.24	0.0
%N2	47.76 ± 7.1	47.44 ± 5.4	46.32 ± 9.0	48.04 ± 7.5	0.0	0.8	1.56
%N3	24.27 ± 4.3	23.67 ± 5.0	24.45 ± 9.5	21.78 ± 6.5	0.54	4.57*	1.85
%REM	25.50 ± 5.2	25.81 ± 4.8	24.67 ± 4.8	25.04 ± 5.2	0.35	0.22	0.01
REM Latency	93.75 ± 35.8	78.71 ± 23.5	103.56 ± 51.5	101.88 ± 55.0	2.39	0.99	0.64
Lights out to N2	17.98 ± 17.1	22.95 ± 13.6	43.16 ± 43.6	43.94 ± 41.2	5.48*	1.06	0.04
Awakenings, number	5.55 ± 4.0	5.68 ± 5.1	8.19 ± 5.5	9.06 ± 5.3	6.41*	0.31	0.0
Awakenings, number per hour of SPT	0.75 ± 0.5	0.79 ± 0.7	1.19 ± 0.6	1.34 ± 0.8	9.99**	0.46	0.01
Stage shifts, number	55.70 ± 17.4	59.47 ± 16.4	69.19 ± 28.1	77.75 ± 23.7	8.12**	1.46	0.17
Stage shifts, number per hour of SPT	7.58 ± 2.3	8.37 ± 1.9	10.69 ± 2.8	11.29 ± 3.1	23.37***	1.08	0.15
REM periods, number	3.90 ± 0.7	3.95 ± 0.7	3.38 ± 1.3	3.75 ± 1.0	2.19	1.97	1.6
REM periods, duration	29.49 ± 6.3	29.73 ± 7.4	32.74 ± 14.7	29.92 ± 10.5	0.43	0.41	0.9
Relative delta power N3	0.87 ± 0.0	0.86 ± 0.0	0.87 ± 0.1	0.86 ± 0.1	0.0	0.0	1.07
Relative delta power N2	0.68 ± 0.0	0.68 ± 0.0	0.74 ± 0.1	0.73 ± 0.1	6.96*	0.0	0.35
Relative delta power REM	0.60 ± 0.1	0.58 ± 0.1	0.65 ± 0.1	0.65 ± 0.1	6.57*	1.71	0.51
Relative sigma power N2	0.05 ± 0.0	0.04 ± 0.0	0.04 ± 0.0	0.04 ± 0.0	2.01	0.0	1.42
Relative theta power REM	0.21 ± 0.0	0.22 ± 0.0	0.18 ± 0.0	0.18 ± 0.0	9.34**	0.0	0.7
Spindles, number	942.55 ± 445.4	878.11 ± 339.6	726.56 ± 382.3	607.88 ± 366.3	3.67	3.79	0.01
Spindles, duration	0.90 ± 0.1	0.90 ± 0.1	0.91 ± 0.1	0.90 ± 0.1	0.06	0.0	1.71
Spindles, frequency	13.64 ± 0.3	13.68 ± 0.3	13.77 ± 0.5	13.80 ± 0.5	0.92	0.84	0.03
Spindles, density	3.11 ± 1.3	3.02 ± 1.1	2.82 ± 1.2	2.22 ± 1.3	1.54	8.69**	3.52
REM alpha bursts, number	82.45 ± 63.2	114.32 ± 109.3	113.19 ± 103.4	161.62 ± 147.4	1.22	4.15*	0.34
REM alpha bursts, density	0.78 ± 0.5	1.06 ± 1.0	1.45 ± 1.5	1.84 ± 1.7	3.73	2.21	0.1
REMs, number (average)	133.00 ± 74.9	110.07 ± 98.0	154.42 ± 73.3	219.04 ± 81.1	6.37*	0.71	5.23*
REMs, density (average)	1.39 ± 0.9	0.98 ± 0.7	1.69 ± 0.6	2.26 ± 0.5	12.16**	0.02	8.26**

Significant F-values are in bold.

*TST* total sleep time, *SPT* sleep period time, *SOL* sleep onset latency, *WASO* wake after sleep onset, *SE* sleep efficiency, *SME* sleep maintenance efficiency.

*p < 0.05, **p < 0.01, ***p < 0.001.

Because our patient and control groups significantly differed in age (see Table 1), we ran the analyses again after adjusting for age. While the interactions for the density and absolute count of REMs remained significant, the interaction for TST and the duration of N2 sleep were no longer significant (p = 0.18 and p = 0.14, respectively). Interestingly, age-adjusted models also revealed a significant interaction for the relative delta and theta power in REM sleep (delta: F(1, 31) = 6.15, p = 0.019; theta: F(1, 31) = 4.41, p = 0.044). Post hoc tests revealed a pattern of significantly higher delta power and lower theta power in EMDR patients compared to controls at T1 (p = 0.007 and p = 0.011, respectively) but not at T0 (both p’s > 0.25).

We also found a main effect of Group (EMDR vs Control) for several sleep parameters. Specifically, compared to control participants, patients in the EMDR group had significantly shorter TST (all F-values are shown in Table 2) and sleep period time (SPT), longer sleep onset latency (SOL), lower sleep efficiency (SE) and sleep maintenance efficiency (SME), longer duration and percentage of N1 sleep and shorter duration of N3 sleep and REM sleep, longer latency from lights out to N2 sleep, higher number of awakenings and stage transitions (both absolute and density per hour of SPT), higher relative delta power in N2 sleep and REM sleep, lower relative theta power in REM sleep, higher number and density of REMs during REM sleep. Age-adjusted models revealed an overall similar pattern of group differences, though 7 out of the above 19 sleep parameters were no longer significantly different between groups: SME, duration of N1 and N3 sleep, number and density of awakenings, number of stage transitions, and relative theta power in REM sleep.

To facilitate the comparison of our findings to Raboni et al. 2014’s study, we report in Table S1 the effect sizes (Cohen d) of the comparison of all sleep parameters, both within each group and between the two groups at each time point.

**Figure 2:**
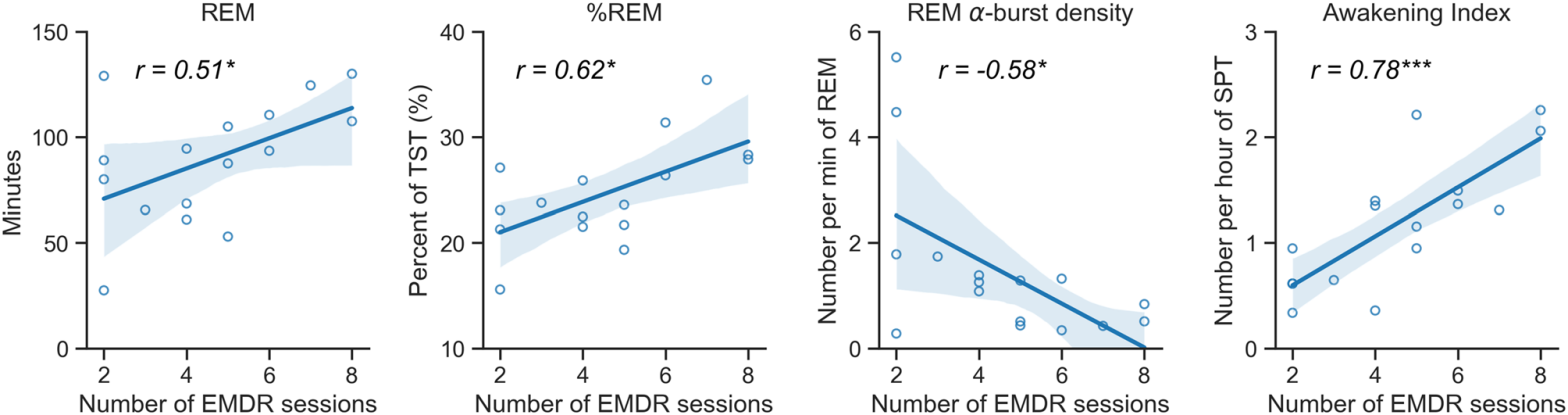
Significant correlations between number of EMDR sessions before remission and sleep parameters before therapy. *p < 0.05, ***p < 0.001.

Having tested the main effects of group as well as the interaction between group and time, we next investigated whether the number of EMDR sessions in the patient group was predicted by sleep parameters at baseline. We found significant correlations between the number of EMDR sessions required to reach remission and the following sleep parameters, the duration and percentage of REM sleep (duration: r = 0.51, p = 0.04; percentage: r = 0.615, p = 0.01), the number and density of awakenings (number: r = 0.68, p = 0.004; density: r = 0.78, p < 0.001), and the density of alpha bursts during REM sleep (r = − 0.58, p = 0.01). With the exception of the latter, higher values for these sleep parameters before therapy predicted a higher number of EMDR sessions required to reach remission (see Fig. 2 and Table S2). Of note, only the density of awakenings remained significant after applying a Holm-Bonferroni correction for multiple comparisons.

We also explored whether the number of EMDR sessions required to reach remission was correlated with sleep parameters after treatment (Table S2). We found that the TST (r = 0.56, p = 0.02), SPT (0.61, p = 0.01) and duration of N2 sleep (0.56, 0.02) after treatment were all significantly positively correlated with the number of EMDR sessions; though none remained significant after Holm-Bonferroni correction.

Finally, we investigated whether the changes in sleep parameters from T0 to T1 were associated with better or worse improvements of PTSD symptoms from T0 to T1. Using double-difference correlations, we found that the participants with the strongest increase in REMs density from T0 to T1 were also the ones with the strongest decrease in depression symptoms measured on the BDI scale (r = − 0.67, p = 0.018). Due to the large number of combinations between sleep parameters and clinical outcomes, this correlation was however not significant after adjusting for multiple comparison. Noteworthy, the above correlation was the only one that was significant (uncorrected) among all the combinations.

## Discussion

We examined the associations between PTSD symptoms remission and sleep parameters in military-related PTSD subjects, and further compared them to a group of healthy military service members. In support of our hypothesis, symptoms remission improved both subjective and objective sleep outcomes. We showed that symptom remission after EMDR therapy was associated with significantly increased REMs number and density in REM sleep (Table 2). To note some expected effects did not survive correction for multiple comparisons: TST and N2 stage duration were increased at T1, and an increased REMs density between T0 and T1 predicted a greater decrease of depression symptoms on the BDI scale. We also observed a significant group effect on several sleep parameters which suggests that the symptoms remission did not completely normalize sleep in the patient group. Finally, we report that some objective sleep measures at T0 are predictive of the number of EMDR therapy sessions required to reach symptoms remission. The number and density of awakenings during SPT (as well as the duration and percentage of REM sleep before treatment, even though not significant after correction for multiple comparisons) was indeed significantly correlated with the number of EMDR sessions needed to reach remission. Specifically, the number of EMDR sessions required for symptoms remission was positively correlated with more awakenings (and more REM sleep, but it did not survive after correction for multiple comparisons) before treatment (Fig. 2 and Table S2).

One week before therapy onset, sleep parameters of PTSD patients were consistent with previous literature on sleep disturbances in PTSD, i.e. shorter TST and N2 sleep, as well as lower subjective sleep quality compared to control individuals. After remission from PTSD symptoms with EMDR therapy, both TST and duration of N2 sleep were no longer significantly different between groups, suggesting a normalization of sleep duration in patients (specifically N2 sleep) and REMs density was significantly increased. These findings are consistent with a recent study on written narrative exposure therapy^83^. In this study, which had no control group, PTSD participants showed longer total sleep time, higher percentage of N2 sleep and higher REMs density after therapy compared to before therapy. However, caution must be exercised when comparing this latter study to the present one since they measured the PSG sleep parameters on the night following therapy, and not one week later as in our study. Our results are also consistent with a previous study that showed an improvement of objective sleep measures in non-military-related PTSD patients treated with EMDR therapy^43^. The sleep parameters that were improved following therapy are however different in the two studies since Raboni et al. reported only a decreased WASO after treatment (here also suggesting a normalization of sleep). A possible explanation for the results discrepancy between studies is the relatively small sample size. More surprisingly however, Raboni et al.^43^ also reported a non-significant higher REMs density before treatment in PTSD patients compared to the control group (Cohen d = 1.05), with little or no between-group difference after treatment (d = 0.16) due to reduction of REM density in PTSD patients, whereas we observed an evolution in the opposite direction i.e. a significantly higher REMs density in the PTSD group compared to the control group after treatment, with no between-group differences before treatment. Our result is in line with the higher REMs density in PTSD patients following written narrative exposure therapy observed by Kobayashi et al.^83^. However, they also reported that increased REM density was negatively associated with PTSD symptom reduction, while we observed the opposite correlation. These differences of results may be due to the small sample size and to the control groups which may vary in their matching with the patients’ characteristics (i.e. age, profession) according to the different studies.

We also found significant group effects for a large number of objective sleep parameters, which are all in the direction of poorer sleep quality in PTSD patients than in the control group (e.g. lower efficiency and N3 sleep duration, higher percentage of N1 sleep, longer sleep latency, higher number of awakenings and stage transitions). In other words, even if remission was associated with some improvements in TST and N2 sleep, PTSD patients did not reach a normalized sleep even when symptoms disappeared, which contrasts with the strong improvement in subjective sleep quality.

Exposure to trauma is associated with an integration of distressing and emotionally charged experiences into one’s autobiographical memory. In PTSD, this lack of integration and depotentiation could be associated with an improper and non-adaptive over-consolidation of traumatic experiences^84^ underlying the intrusive and distressing nature of traumatic memories. It is now well-established that sleep is essential for memory consolidation and emotional regulation of past events^85^. Over the years, accumulated evidence has suggested that REM sleep and NREM sleep may serve distinct and complementary functions in memory consolidation^86^. REM sleep has consistently been shown to support the specific consolidation of emotionally-salient memories^87–90^. By contrast, NREM sleep is thought to support consolidation of non-emotional episodic memory^91^. Specifically, during NREM sleep, relevant new memories are thought to be integrated and reorganized with respect to already existing experiences^7^. In other words, the restoration of N2 sleep after symptoms remission suggested by several studies comprising ours, could restore the ability to integrate the traumatic event into autobiographical memory and thus prevent intrusions of traumatic memories.

On the other hand, REM sleep is thought to be important for depotentiating the affective tone of emotional memories^92^. Specifically, emotional memories are thought to be reactivated during REM sleep by amygdala reactivations, causing a strengthening of the declarative, informational content of the memory and a decrease of the emotional reactivity to this memory^90,92–99^. In line with this, the emotional intensity of waking life memories is attenuated in dreams in healthy subjects^93,96^ but not in PTSD patients for the traumatic event that caused the disorder. In this study, we found that symptoms remission using EMDR increased the density of REMs in REM sleep. Such increase in REMs density may favour traumatic memory integration and depotentiation during sleep as the eye movements of the EMDR therapy may favor this process at wake^55^. Building on the fact that eye movements in both REM sleep and wakefulness activate similar cortical areas^100^, Stickgold proposed that eyes movements could be the process by which emotional depotentiation happens both during REM sleep and EMDR therapy. According to this hypothesis, the alternating bilateral stimuli of EMDR would activate brain mechanisms that shift the brain into a memory processing mode similar to that of REM sleep. This REM-like state would permit the integration of traumatic memories into associative cortical networks without interference from hippocampus-mediated episodic recall. Our results are in line with this hypothesis, notably the correlation (non-significant after correction for multiple comparison) showing that the increased REMs density between T0 and T1 predicted a greater decrease of symptoms on the BDI scale.

Based on these findings and theoretical insights, we therefore propose that the to-be confirmed increase in duration in N2 sleep after symptoms remission could reflect the restored ability of PTSD patients to defend against traumatic memory intrusions, whereas the increase in REMs density could reflect a REM-related regulation mechanism rebound to decrease the emotional load of the traumatic memory.

Another important finding of this study was the positive correlation between the number of EMDR sessions needed to achieve remission in the PTSD group and sleep parameters before therapy, including a positive correlation with the number and density of intra-sleep awakenings (and also the percentage of REM sleep that did not survive after correction for multiple comparisons) (Fig. 2). In other words, PTSD patients with higher density of intra-sleep awakenings and possibly longer REM sleep at baseline were also the ones that needed the more EMDR sessions to reach symptom remission. This finding may indicate that having a great amount of intra-sleep awakenings (and possibly REM sleep) before treatment is maladaptive and/or reflect a failure of REM-sleep emotional regulation mechanisms. Intra-sleep awakenings are known to be positively correlated with dream recall frequency^81,101^ which is correlated with nightmares frequency^102^. Patients with the more intra-sleep awakenings may thus also be the ones with the more nightmare recall, and intra-sleep awakenings may interrupt the regulation process during REM sleep. Bilateral alternating stimulation (BAS) during EMDR therapy could substitute this maladaptive or lack of emotional regulation process normally at play during REM sleep and participate in the emotional memory depotentiation and reconsolidation, via notably mental travel in time^86,103,104^, which also happens during dreaming since very old memories can be part of the dream scenery^93,105^.

In conclusion, our results show that remission from military-related PTSD (with EMDR therapy) is associated with an improvement of some sleep parameters, notably an increase of REMs density in REM sleep and possibly TST and N2 durations. As it is argued in the literature that sleep-related memory consolidation may depend on several inter-dependant sleep stages^86^, it may be the case that remission in PTSD patients is explained by the combination of an increase in N2 duration and REMs density in REM sleep. The increase in REMs density may require improved N2 to be efficient. Future studies are warranted to further test this hypothesis and to improve our understanding of the neurophysiological mechanism responsible for symptom disparition in PTSD patients treated by EMDR therapy.

This is important to note that this study has some limitations. The first limitation is that the control group consisted of healthy individuals without PTSD. Our healthy control group has the advantage of providing us with normative sleep data in healthy subjects from the same profession, thus sharing the same environment and constraints as our patients. One of its limitations is that the mean age of the EMDR group was higher than the one of the control group by an average of seven years, which may have biased group-comparison of sleep duration and quality, both known to decrease with age^106^. Indeed, some of the above-described interactions and group effects were no longer significant when adjusting for age. Future studies with larger sample sizes and age-matched groups are therefore needed to replicate our findings. Another limitation of the study is related to the absence of a wait-list control group to ensure that the simple passage of time does not explain the effects observed, and of a PTSD group treated with another therapy to assess the specificity of the effect observed with the EMDR therapy. Note however that the correlations between sleep parameters and the number of EMDR sessions needed to reach remission speak in favor of the specificity of the EMDR therapy. Importantly, although the study design does not allow to establish whether the sleep changes observed are related to therapy vs the mere passage of time, it is unlikely that the passage of time explains our results. Indeed, according to the DSM5 criteria, PTSD symptoms have to be present for more than one month for the disorder to be actually classified as DSM5. And regarding more specifically the sleep symptoms, a study showed in 1995 that 59 to 73% of Vietnam veterans suffering from PTSD still had sleep disturbances such as insomnia and nonrestorative sleep i.e. in these patients the sleep disturbances had lasted at least for 20 years^32^. Finally, it has been frequently observed in PTSD patients that sleep disturbances do not improve after otherwise successful first-line PTSD treatment^24,26^ and disturbed sleep is one of the two most reported residual symptoms^26^. In line with these clinical observations, from a biological point of view numerous studies have demonstrated the chronic nature of sleep disorders in PTSD. In 2019 Colvonen et al.^107^, demonstrated that sleep disturbances in 40 veterans with PTSD were unremitting without direct intervention, after a 3 months follow-up. In other words, some further studies are needed to confirm our results and verify the specific effect of EMDR therapy on sleep parameters by using a wait-list control group, a non-PTSD-dedicated therapy or care, and another PTSD-dedicated therapy as exposure therapy or cognitive behavioral therapy trauma focused.

Another limitation is that our two groups were exclusively composed of male soldiers—mainly for practical reasons (as explained in the Methods). Our findings should therefore not be generalized to female soldiers and/or non-military populations. PTSD patients under psychotropic medication were included in the analysis if they were taking SSRIs only if the medication had stayed consistent for at least three months prior to and throughout the trial. Recent meta-analyses showed that SSRIs may increase REM latency, suppress REM sleep and impair sleep continuity^108,109^. At inclusion, however, the intensity of symptoms measured by the CAPS or the duration of REM did not differ between individuals taking SSRIs and those without psychotropic drugs.

Finally, the polysomnography recording devices were different between our two groups, and this may have artificially driven group differences in EEG-based spectral power and/or EOG-based REMs number and density. We cannot exclude that some of the group differences observed in sleep parameters could be due to be different recording setting and experimental procedure for controls and patients, i.e. in-lab PSG with a constrained time in bed for the control group, versus at-home ambulatory PSG with no time constraint for the patient group. However, this limitation cannot explain the time effect found in patients.

In summary, our findings show that remission from military-related PTSD with EMDR therapy is associated with an increase in REMs density during REM sleep and possibly with a normalization of total sleep duration, mostly driven by an increase of N2 sleep. However, a wait-list group and another therapy PTSD therapy group is needed to confirm these results, and the specificity of the EMDR therapy on these effects. Furthermore, we show that the number and density of intra-sleep awakenings before treatment are both predictive of the number of EMDR sessions required to reach remission.

## Supplementary Information

Supplementary Information.

## Data Availability

Data is available on request from the corresponding author.

## Abbreviations

EMDR: Eye movement desensitization and reprocessing
PSG: Polysomnography
PTSD: Posttraumatic stress disorder
TST: Total sleep time
SPT: Sleep period time
SOL: Sleep onset latency
REM: Rapid eye movement
SE: Sleep efficiency
SME: Sleep maintenance efficiency
WASO: Wake after sleep onset
