# Supplementary material for: Sleep parameters improvement in PTSD soldiers after symptoms remission
Source: Sci Rep. 2021 Apr 23;11:8873. doi: 10.1038/s41598-021-88337-x (PMC8065125; doi:10.1038/s41598-021-88337-x)
Supplement: Supplementary file 1 — Supplementary Information. [file 41598_2021_88337_MOESM1_ESM.docx]

Remission from posttraumatic stress disorder with EMDR therapy is associated with improved sleep parameters in soldiers

Rousseau PF^1*^, Vallat R^2,3^, Coste O^4^, Cadis H^1^, Nicolas F^5^, Trousselard M^6^, Ruby P^2,^ Khalfa S^1^

1: Laboratoire de Neurosciences Sensorielles et Cognitives, Aix Marseille Université CNRS, Marseille, France

2: Lyon Neuroscience Research Center, Brain Dynamics and Cognition Team, INSERM UMR 1028, CNRS UMR 5292, Université Claude Bernard Lyon 1, France

3: Center for Human Sleep Science, Department of Psychology, University of California, Berkeley, CA 94720-1650, USA

4: Unité de pathologie du sommeil, Hôpital d’Instruction des Armées Desgenettes, Lyon, France

5: Service de psychiatrie, Hôpital d’Instruction des Armées Sainte-Anne, Toulon, France

6: Unité Neurophysiologie du stress, département des neurosciences et des contraintes opérationnelles, Institut de Recherche Biomédicale des Armées, Brétigny sur Orge, France

**Corresponding author:**

Pierre-François Rousseau : rousseaupierrefrancois@gmail.com

Laboratoire de Neurosciences Sensorielles et Cognitives, Aix Marseille Université CNRS, Marseille, France.

# Supplementary results

**Table S1. Effect sizes of all pairwise comparisons.** To facilitate the comparison of our findings to Raboni et al 2014’s study, we report the effect sizes (Cohen d) of all pairwise comparisons of sleep parameters, both within each group and between the two groups at each time point. Effect sizes greater than 0.5 are highlighted in bold. Negative effect sizes for within-group comparisons mean lower values at T0 than T1. Negative effect sizes for between-group comparisons mean lower values in the EMDR group compared to the control group.

|  | **Within-group** | | **Between-group** | |
| --- | --- | --- | --- | --- |
| **Sleep parameters** | **PTSD** | **Control** | **T0** | **T1** |
| TST | **-0.63** | 0.30 | **-1.04** | **-0.54** |
| SPT | -0.46 | 0.49 | **-0.77** | -0.31 |
| SOL | -0.06 | -0.28 | **0.79** | **0.70** |
| WASO | 0.45 | 0.30 | 0.42 | **0.71** |
| SE | -0.39 | 0.03 | **-1.17** | **-1.11** |
| SME | -0.29 | -0.36 | -0.43 | **-0.72** |
| N1 | -0.31 | -0.38 | **0.59** | **0.75** |
| N2 | **-0.65** | 0.18 | **-0.77** | -0.25 |
| N3 | 0.08 | 0.39 | **-0.80** | **-0.63** |
| REM | -0.4 | -0.03 | **-0.70** | -0.35 |
| %N1 | -0.19 | -0.38 | **0.80** | **0.85** |
| %N2 | -0.35 | 0.04 | -0.18 | 0.09 |
| %N3 | 0.50 | 0.21 | 0.03 | -0.33 |
| %REM | -0.08 | -0.12 | -0.17 | -0.15 |
| REM Latency | 0.02 | **0.51** | 0.23 | **0.57** |
| Lights out to N2 | -0.07 | -0.30 | **0.80** | **0.71** |
| Awakenings, number | -0.10 | -0.15 | **0.56** | **0.66** |
| Awakenings, number per hour of SPT | -0.11 | -0.19 | **0.78** | **0.76** |
| Stage shifts, number | -0.32 | -0.25 | **0.59** | **0.91** |
| Stage shifts, number per hour of SPT | -0.13 | -0.40 | **1.23** | **1.16** |
| REM periods, number | -0.46 | -0.07 | **-0.51** | -0.23 |
| REM periods, duration | 0.30 | -0.06 | 0.30 | 0.02 |
| Relative delta power N3 | 0.05 | 0.14 | -0.01 | -0.06 |
| Relative delta power N2 | 0.08 | 0.04 | **1.01** | **0.85** |
| Relative delta power REM | 0.09 | 0.28 | **0.86** | **0.96** |
| Relative sigma power N2 | 0.11 | 0.17 | -0.49 | **-0.54** |
| Relative theta power REM | -0.01 | -0.21 | **-0.97** | **-1.17** |
| Spindles, number | 0.24 | 0.21 | **-0.52** | **-0.77** |
| Spindles, duration | 0.16 | -0.07 | 0.16 | -0.04 |
| Spindles, frequency | -0.09 | -0.11 | 0.33 | 0.30 |
| Spindles, density | 0.42 | 0.11 | -0.23 | **-0.66** |
| REM alpha bursts, number | -0.44 | -0.36 | 0.37 | 0.37 |
| REM alpha bursts, density | -0.26 | -0.35 | **0.63** | **0.57** |
| REMs, number (average) | **-1.01** | 0.50 | 0.37 | **1.95** |
| REMs, density (average) | **-0.84** | 0.27 | 0.29 | **1.20** |

TST: total sleep time, SPT: sleep period time, SOL: sleep onset latency, WASO: wake after sleep onset, SE: sleep efficiency, SME: sleep maintenance efficiency

**Table S2. Correlations between the number of EMDR sessions required to reach remission and sleep parameters before (T0) and after (T1) EMDR therapy in the PTSD group.** Sleep variables are sorted by p-values at T0 in ascending order**.** Significant uncorrected p-values are in bold. Stars indicate significance after Holm-Bonferroni correction for multiple comparisons. *p<0.05.

| **Time** | **T0** | | **T1** | |
| --- | --- | --- | --- | --- |
| **Sleep variable** | **Pearson r** | ***p*** | **Pearson r** | ***p*** |
| TST | 0.269 | *0.314* | 0.565 | ***0.023*** |
| SPT | 0.256 | *0.339* | 0.612 | ***0.012*** |
| SOL | 0.39 | *0.136* | 0.316 | *0.233* |
| WASO | 0.343 | *0.193* | 0.089 | *0.742* |
| SE | 0.173 | *0.522* | 0.007 | *0.979* |
| SME | -0.11 | *0.685* | 0.121 | *0.656* |
| N1 | 0.281 | *0.293* | 0.088 | *0.746* |
| N2 | 0.064 | *0.813* | 0.56 | ***0.024*** |
| N3 | 0.004 | *0.989* | -0.08 | *0.769* |
| REM | 0.511 | *0.043* | 0.315 | *0.235* |
| %N1 | 0.103 | *0.703* | -0.074 | *0.787* |
| %N2 | -0.186 | *0.490* | 0.278 | *0.297* |
| %N3 | -0.173 | *0.521* | -0.342 | *0.194* |
| %REM | 0.615 | ***0.011*** | 0.072 | *0.792* |
| REM Latency | 0.003 | *0.991* | -0.106 | *0.696* |
| Lights out to N2 | 0.405 | *0.119* | 0.324 | *0.221* |
| Awakenings, number | 0.682 | ***0.004*** | -0.077 | *0.776* |
| Awakenings, number per hour of SPT | 0.781 | ***<0.001**** | -0.182 | *0.501* |
| Stage shifts, number | 0.479 | *0.061* | 0.176 | *0.514* |
| Stage shifts, number per hour of SPT | 0.369 | *0.159* | -0.068 | *0.802* |
| REM periods, number | 0.114 | *0.674* | 0.396 | *0.129* |
| REM periods, duration | 0.175 | *0.516* | 0.018 | *0.947* |
| Relative delta power N3 | 0.185 | *0.493* | 0.109 | *0.689* |
| Relative delta power N2 | 0.359 | *0.172* | 0.17 | *0.530* |
| Relative delta power REM | 0.244 | *0.363* | 0.005 | *0.985* |
| Relative sigma power N2 | -0.229 | *0.394* | -0.302 | *0.255* |
| Relative theta power REM | -0.221 | *0.410* | -0.107 | *0.694* |
| Spindles, number | -0.219 | *0.416* | -0.17 | *0.529* |
| Spindles, duration | -0.218 | *0.417* | -0.316 | *0.234* |
| Spindles, frequency | 0.058 | *0.832* | 0.288 | *0.279* |
| Spindles, density | -0.218 | *0.417* | -0.255 | *0.340* |
| REM alpha bursts, number | -0.437 | *0.091* | -0.224 | *0.403* |
| REM alpha bursts, density | -0.581 | ***0.018*** | -0.237 | *0.377* |
| REMs, number (average) | -0.177 | *0.582* | 0.334 | *0.288* |
| REMs, density (average) | 0.278 | *0.381* | 0.395 | *0.203* |

TST: total sleep time, SPT: sleep period time, SOL: sleep onset latency, WASO: wake after sleep onset, SE: sleep efficiency, SME: sleep maintenance efficiency
